# MAF-Net: A Lightweight Morphology-Adaptive Feature Fusion Network for Semantic Segmentation

**DOI:** 10.3390/s26206593

**Published:** 2026-10-17

**Authors:** Wenze Shi, Ping Liu, Takeshi Ikenaga, Daiki Nobayashi

**Affiliations:** 1 Guangling College, Yangzhou University, Yangzhou 225009, China; 2 Kyushu Institute of Technology, Kitakyushu 8048550, Japan; ike@ecs.kyutech.ac.jp (T.I.); nova@ecs.kyutech.ac.jp (D.N.); 3 School of Information and Artificial Intelligence, Yangzhou University, Yangzhou 225009, China; pliu@yzu.edu.cn

**Keywords:** semantic segmentation, lightweight network, morphology-adaptive kernel, feature fusion, boundary refinement

## Abstract

Semantic segmentation enables pixel-level scene understanding, and deploying it on resource-constrained platforms such as unmanned aerial vehicles and embedded sensing devices has become increasingly important. In complex scenes, however, objects with substantial scale variation and heterogeneous morphology often lead to missed small objects, discontinuities along slender structures, and incomplete boundaries. To address the challenges, this paper proposes a lightweight semantic segmentation network, named MAF-Net. The network adopts MobileNet as the backbone and enhances the representation of objects with different scales and morphological characteristics through multi-scale feature extraction and top-down feature fusion, while maintaining low model complexity. To further accommodate variations in object scale and structure, a Morphology-Adaptive Kernel Decoder (MAKD) is designed. The module introduces large-kernel, small-kernel, horizontal-strip, and vertical-strip convolution branches, and adaptively fuses the multi-branch features through a gating mechanism, thereby providing complementary contextual representations with different receptive-field scales and directional sensitivities. In addition, a Triple Feature Fusion Refinement Module (TFFRM) is constructed to further refine the initial segmentation results by integrating shallow spatial details, learned boundary-aware responses, and high-level semantic features, thereby alleviating problems such as discontinuities in slender structures and blurred object boundaries. Experimental results show that the proposed method achieves favorable overall segmentation performance on the COCO2017-derived ball-sports dataset, UDD6, and WHDLD, while maintaining a relatively small parameter count and high measured inference speed.

## 1. Introduction

Semantic segmentation aims to predict the category of each pixel and assign semantic labels to different regions, thereby providing dense scene understanding at the pixel level [1]. Compared with image classification and object detection, semantic segmentation not only identifies object categories but also describes their spatial extent, contours, and structural details, making it particularly suitable for fine-grained scene understanding [2].

Traditional semantic segmentation methods mainly rely on low-level image cues and probabilistic graphical models. Gray-level segmentation [3] and threshold-based segmentation [4] partition images according to intensity, color, or texture, while Conditional Random Fields (CRFs) [5] and graph-cut-based methods [6] further incorporate spatial consistency and region-boundary constraints. Although these methods are computationally simple and interpretable, their dependence on handcrafted features and manually designed priors limits their semantic representation and generalization ability in complex scenes [7,8].

With the development of deep learning, semantic segmentation has shifted toward end-to-end feature learning [9,10]. CNN-based methods established the foundation of modern semantic segmentation. FCN [11] enabled end-to-end dense prediction, while PSPNet [12] and DeepLabV3 [13] enhanced multi-scale context modeling through pyramid pooling and atrous spatial pyramid pooling, respectively. DeepLabV3+ [14] further introduced an encoder–decoder structure to recover low-level spatial details, whereas UPerNet [15] and CF-Net [16] strengthened hierarchical feature fusion across multiple stages. These approaches are effective for contextual aggregation, but their multi-scale representations are mainly constructed through predefined receptive fields or fixed fusion pathways, which may be less flexible when regions with substantially different scales and geometric structures coexist.

Transformer-based methods further improve global dependency modeling. SETR [17] formulates semantic segmentation as a sequence-to-sequence prediction problem, while SegFormer [18] adopts a hierarchical Transformer encoder for efficient multi-scale representation. Mask2Former [19] introduces masked attention for flexible segmentation modeling, and SegNeXt [20] combines convolutional attention with multi-scale context aggregation. Compared with conventional CNNs, these methods provide stronger global modeling capability; however, the increased computational and memory costs can restrict their application in resource-constrained or real-time scenarios.

More recently, state-space-based vision models have provided another direction for efficient long-range modeling. VMamba [21] and SegMAN [22] exploit selective state-space modeling to capture long-range dependencies, while Vision Mamba [23] introduces bidirectional state-space modeling for visual representation learning. Although these methods improve global context modeling efficiency, their primary focus remains long-range representation rather than explicit adaptation to heterogeneous region morphology. In complex scenes, large-area regions, small objects, elongated structures, and irregular boundaries often coexist. This motivates a segmentation framework that can simultaneously account for scale variation, directional structure, local boundary details, and computational efficiency.

### 1.1. Motivation

Encoder–decoder architectures remain widely used in semantic segmentation because they provide a natural mechanism for combining high-level semantic information with low-level spatial details [24,25]. EDSF-Net [26] improves feature representation through a self-calibrated encoder–decoder design, while Conv-UNet [27] incorporates ConvNeXt into a U-Net-style hierarchy and enhances spatial feature interaction. These studies demonstrate the effectiveness of hierarchical feature reconstruction [28]; however, feature fusion alone does not explicitly address the large morphological differences among semantic regions.

Multi-scale modeling provides a complementary solution. TSG [29] adaptively selects and fuses multi-scale features, CGRSeg [30] employs pyramid context extraction, and CCASeg [31] captures local and global context through convolutional cross-attention. Such methods improve scale robustness compared with single-scale representations [32]. Nevertheless, scale variation is only one aspect of morphology heterogeneity. Narrow roads, elongated structures, small objects, and irregular boundaries may require not only different receptive-field sizes but also different directional sensitivities. Therefore, an adaptive mechanism capable of selecting complementary scale- and direction-aware responses is desirable.

Efficiency is another important consideration for practical deployment. MobileNet [33] and EfficientNet [34] reduce computational cost through lightweight backbone design, while YOLO11m-seg [35] emphasizes efficient localization and mask prediction. TinyViM-S [36] further explores compact architectures for efficient visual modeling. Recent studies in intelligent driving also highlight the importance of adaptive feature interaction. BANet [37] combines bidirectional feature aggregation with adaptive multi-scene perception to enhance hierarchical feature fusion under varying driving conditions. MRGD-Net [38] employs multi-scale feature reconstruction and gated dual attention to strengthen cross-level feature interaction and adaptively emphasize informative regions. Although these methods are designed for lane detection and driver attention prediction, respectively, they demonstrate the effectiveness of adaptive multi-level feature aggregation. However, such strategies do not explicitly address the morphology heterogeneity encountered in semantic segmentation. Therefore, the key challenge is not only to reduce computational complexity, but also to preserve effective representations of small objects, slender structures, and irregular boundaries under a limited computational budget.

Motivated by these observations, our design focuses on two complementary aspects: morphology-adaptive feature modeling and structural refinement. MAKD introduces convolutional branches with different receptive-field scales and directional patterns and adaptively weights them according to spatial content, enabling more flexible modeling of semantic regions with heterogeneous scales and geometric structures. TFFRM further integrates shallow spatial details, boundary-aware responses, and high-level semantic information to refine structural details and object boundaries. In this way, MAF-Net aims to achieve a better balance among morphology-aware representation, structural preservation, and lightweight inference within a unified semantic segmentation framework.

### 1.2. Contributions

Based on the encoder–decoder framework, this paper adopts a lightweight backbone network for multi-scale feature extraction and further designs a Morphology-Adaptive Kernel Decoder and a Triple Feature Fusion Refinement Module. The main contributions of this work are summarized as follows:

1. To reduce the computational cost of semantic segmentation, we propose MAF-Net, a lightweight MobileNetV3-Large-based network that combines multi-scale feature extraction with top-down fusion for efficient prediction.
2. To address missed small targets and discontinuities in slender structures, we design MAKD with large-kernel, small-kernel, horizontal-strip, and vertical-strip convolution branches, adaptively weighted by a spatial gating mechanism.
3. To improve blurred or incomplete object boundaries, we introduce TFFRM, which fuses shallow spatial details, boundary-aware responses, and high-level semantic features to refine segmentation predictions.

## 2. Proposed Method

### 2.1. Overall Architecture of MAF-Net

**Figure 1:**
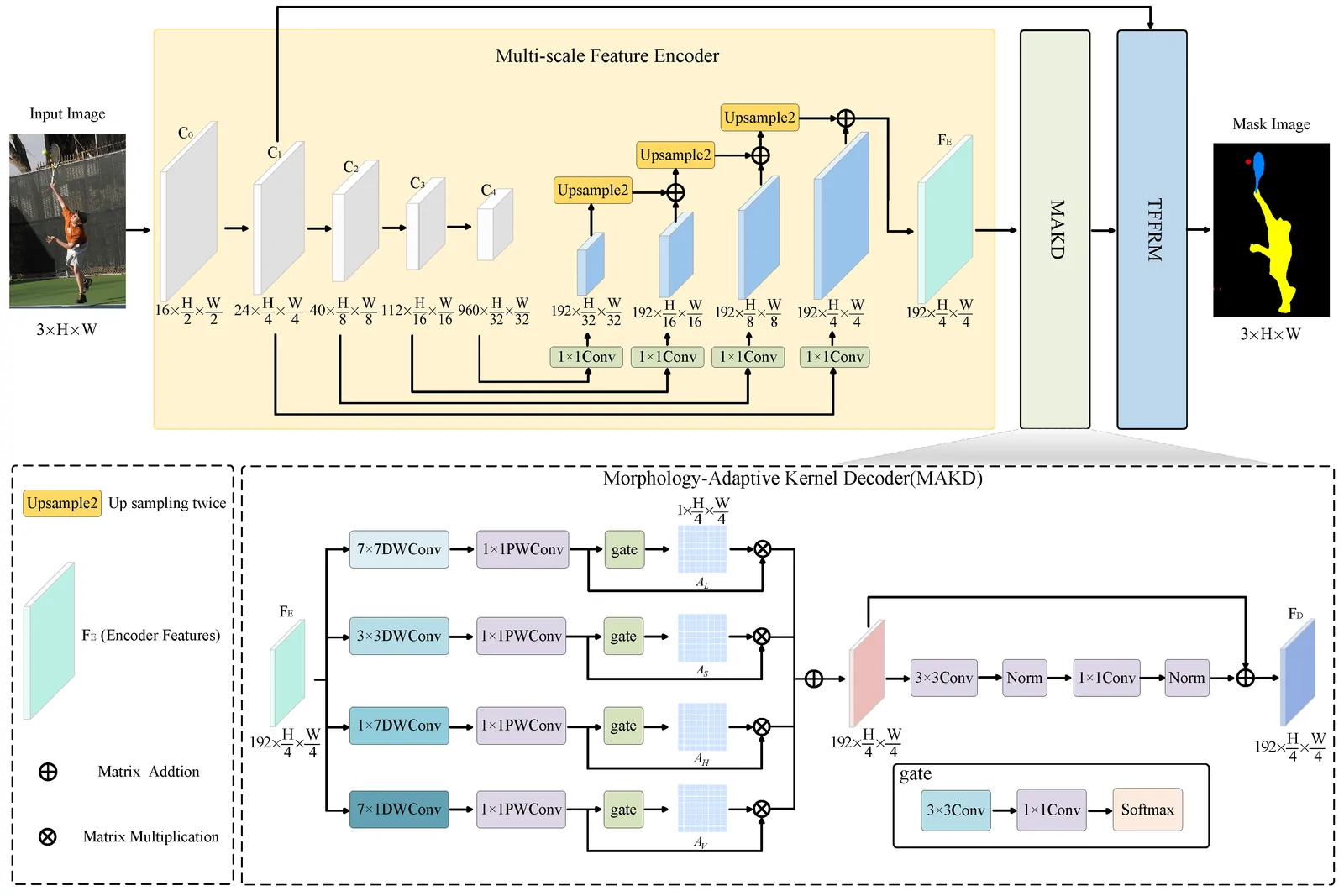
Overall architecture of the proposed MAF-Net. The network uses MobileNetV3-Large to extract multi-scale features and restores high-resolution representations through top-down feature fusion. The MAKD module adaptively captures morphology-aware features with directional convolution branches, while the TFFRM module integrates boundary cues and semantic features to refine coarse segmentation results, producing more accurate segmentation maps. Here, “Norm” denotes Batch Normalization (BatchNorm2d).

As shown in Figure 1, MAF-Net adopts a lightweight encoder–decoder architecture. It mainly consists of three modules: multi-scale feature encoding, morphology-adaptive convolutional decoding, and triple feature fusion refinement module. The following sections describe these modules in detail.

### 2.2. Multi-Scale Feature Encoding

First, the input image is fed into the MobileNetV3-Large backbone to extract feature representations at different scales. The shallow features preserve rich spatial details, while the deep features contain stronger high-level semantic information. Then, a top-down feature fusion strategy is adopted, where high-level semantic features are progressively upsampled and fused with low-level features. In this way, the encoded feature $F_{E}$ with both semantic representation capability and spatial resolution is obtained. The process is formulated as follows:

(1) $$\left\{\begin{array}{c} F_{0}={\mathrm{Conv}}_{1\times 1}\left(C_{4}\right),\\ F_{i}={\mathrm{Conv}}_{1\times 1}\left(C_{4-i}\right)+\mathrm{Up}\left(F_{i-1}\right),\;i=1,2,3,\\ F_{E}=F_{3}. \end{array}\right.$$

where $C_{i}$ denotes the feature maps extracted from different levels of the MobileNetV3-Large backbone, and $F_{i}$ represents the intermediate feature obtained after the *i*-th top-down fusion. ${\mathrm{Conv}}_{1\times 1}$ denotes a 1×1 convolution used to project features into the same channel dimension, and Up(·) denotes the upsampling operation in the spatial dimension.

### 2.3. Morphology-Adaptive Kernel Decoder

As shown at the bottom of Figure 1, we design a Morphology-Adaptive Kernel Decoder (MAKD) to enhance the representation of semantic regions with heterogeneous scales and geometric structures. In complex segmentation scenes, large-area regions may coexist with small objects, elongated structures, and irregularly shaped regions. For example, the ball-sports dataset contains large athlete regions, small ball targets, and slender sports equipment, while aerial and remote-sensing scenes contain narrow roads, small vehicles, buildings, vegetation, and other regions with diverse spatial extents and geometric patterns. A single-scale or fixed-shape convolution kernel cannot effectively model these morphological variations. To address this issue, MAKD introduces multiple convolution branches with complementary receptive-field scales and directional sensitivities, enabling adaptive feature modeling for semantic regions with different morphological characteristics.

MAKD first adopts large-kernel, small-kernel, horizontal strip, and vertical strip depthwise convolutions, followed by pointwise convolutions, to extract contextual information of different morphologies from $F_{E}$. The process is defined as follows:

(2) $$\left\{\begin{array}{c} F_{L}={\mathrm{PWConv}}_{1\times 1}\left({\mathrm{DWConv}}_{7\times 7}\left(F_{E}\right)\right),\\ F_{S}={\mathrm{PWConv}}_{1\times 1}\left({\mathrm{DWConv}}_{3\times 3}\left(F_{E}\right)\right),\\ F_{H}={\mathrm{PWConv}}_{1\times 1}\left({\mathrm{DWConv}}_{1\times 7}\left(F_{E}\right)\right),\\ F_{V}={\mathrm{PWConv}}_{1\times 1}\left({\mathrm{DWConv}}_{7\times 1}\left(F_{E}\right)\right). \end{array}\right.$$

where DWConv(·) and PWConv(·) denote depthwise convolution and pointwise convolution, respectively. $F_{L}$ is intended to capture broader contextual information, while $F_{S}$ emphasizes relatively local spatial patterns. $F_{H}$ and $F_{V}$ model contextual information along the horizontal and vertical directions, respectively. These branches therefore provide complementary feature responses with different receptive-field scales and directional sensitivities.

To enable the network to adaptively weight the complementary convolutional branches at different spatial locations, gated weights are further introduced. Specifically, the gated weight generation function G(·) consists of a 3×3 convolution followed by a 1×1 convolution:

(3) $$G\left(F_{E}\right)={\mathrm{Conv}}_{1\times 1}\left({\mathrm{Conv}}_{3\times 3}\left(F_{E}\right)\right),$$

where $F_{E}\in R^{C\times H/4\times W/4}$ and C=192. Therefore, $G\left(F_{E}\right)\in R^{4\times H/4\times W/4}$ contains four branch-wise logits corresponding to the large-kernel, small-kernel, horizontal-strip, and vertical-strip branches, respectively.

The adaptive gating weights are then obtained by applying Softmax along the branch dimension:

(4) $$A={\mathrm{Softmax}}_{\mathrm{branch}}\left(G(F_{E})\right),\;A=\left[A_{L},A_{S},A_{H},A_{V}\right],$$

where $A_{L}$, $A_{S}$, $A_{H}$, and $A_{V}$ $\in R^{1\times H/4\times W/4}$ denote the spatially adaptive weights of the four convolutional branches. At each spatial location, the four weights satisfy $A_{L}+A_{S}+A_{H}+A_{V}=1$.

Finally, the multi-branch features are weighted and fused under the guidance of the gated weights. Then, convolutional enhancement is applied to further integrate spatial and channel information. The enhanced feature is added to the fused feature through a residual connection to obtain the decoded feature $F_{D}$:

(5) $$F_{D}=A_{L}F_{L}+A_{S}F_{S}+A_{H}F_{H}+A_{V}F_{V}+\mathrm{Enh}\left(A_{L}F_{L}+A_{S}F_{S}+A_{H}F_{H}+A_{V}F_{V}\right).$$

where Enh(·) denotes the convolutional enhancement operation.

Through this module, the network can assign different weights to complementary receptive-field and directional features at each spatial location. This provides a flexible decoding mechanism for scenes containing objects with diverse scales and geometric structures.

### 2.4. Triple Feature Fusion Refinement Module

**Figure 2:**
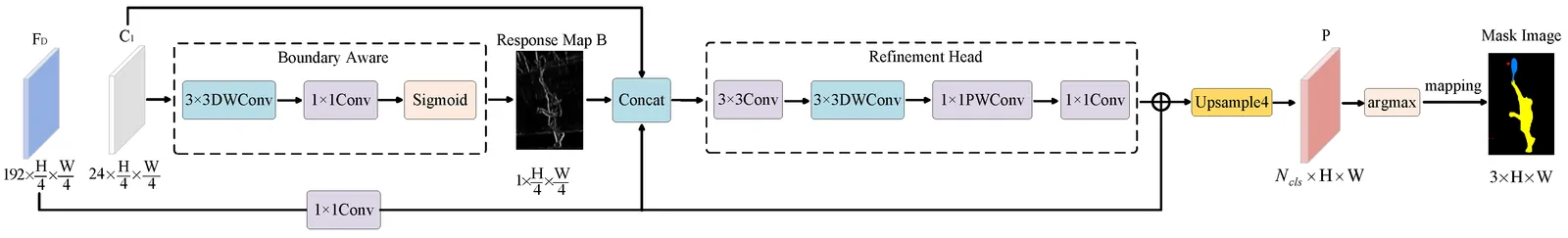
TFFRM Module Details.

As shown in Figure 2, the decoded feature $F_{D}$ and the shallow feature $C_{1}$ are fed into the Triple Feature Fusion Refinement Module (TFFRM). Since shallow features preserve richer spatial details, edge responses, and local structural information, $C_{1}$ is first processed by a lightweight boundary-aware response branch composed of a 3×3 depthwise convolution, a 1×1 convolution, and a Sigmoid activation. The resulting spatial response map *B* is expressed as

(6) $$B=\sigma \left({\mathrm{Conv}}_{1\times 1}\left({\mathrm{DWConv}}_{3\times 3}\left(C_{1}\right)\right)\right),$$

where σ(·) denotes the Sigmoid function. Unlike explicitly supervised boundary prediction, *B* is learned implicitly through the final semantic segmentation objective and serves as a boundary-aware structural cue for feature refinement.

Then, the learned response map *B*, the shallow feature $C_{1}$, and the decoder feature projected by a 1×1 convolution are concatenated. The concatenated feature is further processed by a refinement branch composed of standard convolution, depthwise convolution, and pointwise convolution. Meanwhile, a residual connection is introduced by mapping $F_{D}$ through a 1×1 convolution and adding it to the refined feature. This operation preserves the original semantic information while incorporating shallow spatial details and boundary-aware structural cues.

Finally, the boundary response *B*, the shallow feature $C_{1}$, and the projected decoder feature are concatenated for feature refinement. Let

(7) $$X=\mathrm{Concat}\left(B,\;C_{1},\;{\mathrm{Conv}}_{1\times 1}\left(F_{D}\right)\right).$$

According to the refinement head shown in Figure 2, the refinement operation is explicitly defined as

(8) $$\mathrm{Refine}\left(X\right)={\mathrm{Conv}}_{1\times 1}\left({\mathrm{PWConv}}_{1\times 1}\left({\mathrm{DWConv}}_{3\times 3}\left({\mathrm{Conv}}_{3\times 3}\left(X\right)\right)\right)\right),$$

where the 3×3 standard convolution first performs feature fusion, the 3×3 depthwise convolution further captures local spatial information, and the 1×1 pointwise convolution performs channel interaction. The final 1×1 convolution projects the refined feature to $N_{\mathrm{cls}}$ class logits.

The refined prediction logits are then combined with the projected decoder feature through a residual connection and upsampled to the original image resolution:

(9) $$\;Z=\mathrm{Up}\left[\mathrm{Refine}\left(X\right)+{\mathrm{Conv}}_{1\times 1}\left(F_{D}\right)\right].$$

A Softmax operation is subsequently applied along the class dimension to obtain the pixel-wise semantic probability map *P*:

(10) $$P_{i,k}=\frac{\exp\left(Z_{i,k}\right)}{\sum_{j=1}^{N_{\mathrm{cls}}}\exp\left(Z_{i,j}\right)}.$$

The final semantic segmentation mask is generated by selecting the class with the maximum probability at each pixel:

(11) $$M_{i}=\mathrm{Mapping}\left(\underset{k}{\arg\max}\;P_{i,k}\right).$$

Here, Concat(·) denotes feature concatenation, Up(·) denotes spatial upsampling, and Mapping(·) converts the predicted class indices into the corresponding semantic labels. *Z* denotes the final class-logit map, and $P_{i,k}$ represents the predicted probability that pixel *i* belongs to semantic class *k*.

### 2.5. Loss Function

Based on the probability map *P* defined in Equation (10), let $P_{i,k}$ denote the predicted probability that pixel *i* belongs to semantic class *k*, where $k\in \{1,2,\ldots ,N_{\mathrm{cls}}\}$. The ground-truth label of pixel *i* is denoted by $g_{i}$, and its one-hot representation is denoted by $G_{i,k}$. The set of valid pixels is denoted by Ω.

Considering the class imbalance among different semantic categories, weighted cross-entropy loss is adopted for pixel-wise semantic classification:

(12) $$L_{\mathrm{ce}}=-\frac{1}{\left|\Omega \right|}\sum_{i\in \Omega }\omega_{g_{i}}\log P_{i,g_{i}},$$

where $\omega_{g_{i}}$ denotes the class weight corresponding to the ground-truth category $g_{i}$.

To further improve the region-level overlap between the prediction and ground-truth mask, the multi-class Dice loss is employed:

(13) $$L_{\mathrm{dice}}=1-\frac{1}{N_{\mathrm{cls}}}\sum_{k=1}^{N_{\mathrm{cls}}}\frac{2\sum_{i\in \Omega }P_{i,k}G_{i,k}+\epsilon }{\sum_{i\in \Omega }P_{i,k}+\sum_{i\in \Omega }G_{i,k}+\epsilon },$$

where ϵ is a smoothing factor introduced for numerical stability.

The overall training objective is defined as

(14) $$L=L_{\mathrm{ce}}+\lambda_{\mathrm{dice}}L_{\mathrm{dice}},$$

where $\lambda_{\mathrm{dice}}$ controls the contribution of the Dice loss. In our experiments, $\lambda_{\mathrm{dice}}$ is set to 1.

### 2.6. Overall Algorithm of MAF-Net

To provide a clear overview of the complete inference process, the overall procedure of MAF-Net is summarized in Algorithm 1.
**Algorithm 1** MAF-Net**Require:** 
Input image $I\in R^{3\times H\times W}$.**Ensure:** 
Predicted semantic segmentation mask $M\in R^{H\times W}$.  1:Feed the input image *I* into MobileNetV3-Large to extract multi-scale features, and perform top-down feature fusion according to Equation (1) to obtain the encoded feature $F_{E}$.  2:Send $F_{E}$ into the MAKD module. Extract the four morphology-aware branch features, generate the adaptive gating weights, and perform weighted feature fusion and convolutional enhancement according to Equations (2)–(5) to obtain the decoded feature $F_{D}$.  3:Generate the boundary-aware response *B* from the shallow feature $C_{1}$, and fuse *B*, $C_{1}$, and $F_{D}$ through TFFRM according to Equations (6)–(9) to obtain the refined class-logit map *Z*.  4:Apply Softmax along the class dimension according to Equation (10) to obtain the pixel-wise semantic probability map *P*.  5:Generate the final semantic segmentation mask *M* according to Equation (11).

## 3. Experiments and Results

### 3.1. Dataset Description

One of the datasets used in the experiments was re-screened and reconstructed based on the COCO2017 dataset [39] for semantic segmentation in ball-sport scenes. The original COCO2017 dataset contains approximately 118,000 images in the training set and 5000 images in the validation set, covering various object categories such as persons, vehicles, and sports equipment. It is characterized by diverse object categories, complex scenes, and significant variations in object scale and has therefore been widely used in computer vision tasks such as object detection, image segmentation, and scene understanding.

In this work, the instance-level annotations provided by the COCO dataset were used to re-screen and reorganize the data. First, images containing both the “person” and “sports ball” categories were selected as the basic samples. The person regions were uniformly mapped to the “athlete” category, while sports balls were mapped to the “ball” category. In addition, to enhance the model’s ability to recognize sports equipment, several related categories, including “baseball bat”, “baseball glove”, and “tennis racket”, were retained and uniformly mapped to the “equipment” category, making the reconstructed dataset more focused on sports scenes. After screening, the final dataset contains 4165 images, including 3332 training images, 416 validation images, and 417 test images. The dataset consists of four semantic categories: “background”, “athlete”, “ball”, and “equipment”. This reorganization effectively reduces the interference caused by task-irrelevant categories while emphasizing representative segmentation targets such as athletes, small-scale ball objects, and slender sports equipment.

In addition, to further evaluate the applicability of the proposed model across different scene types, the six-class version of the Urban Drone Dataset, UDD6 [40], was also employed in the experiments. UDD is a high-resolution urban image dataset collected from unmanned aerial vehicle (UAV) platforms in multiple cities and regions. It mainly contains complex urban objects such as roads, buildings, vegetation, and vehicles. The dataset exhibits large variations in object scale, complex spatial layouts, and significant changes in class boundaries, making it suitable for evaluating the feature extraction and boundary modeling capabilities of semantic segmentation models in complex aerial scenes. The UDD6 dataset contains six semantic categories, namely “Facade”, “Road”, “Vegetation”, “Vehicle”, “Roof”, and “Other”.

The UDD6 dataset typically contains 141 high-resolution RGB aerial images, of which 106 images are used for training, while the original validation set of 35 images is further divided into 17 validation images and 18 test images. The image resolutions mainly include 3840×2160, 4096×2160, and 4000×3000. Compared with the ball-sport scene dataset, UDD6 differs significantly in terms of object categories, viewpoints, and spatial structures. Therefore, UDD6 is employed as a cross-scene benchmark to evaluate whether the proposed morphology-adaptive design remains effective under aerial viewpoints, urban object distributions, and spatial structures that differ substantially from those of the ball-sports dataset.

Furthermore, the Wuhan Dense Labeling Dataset (WHDLD) [41,42] is introduced as a second cross-scene benchmark to further assess the applicability of the proposed model to remote-sensing land-cover segmentation. WHDLD is a densely annotated remote sensing image dataset constructed from satellite imagery covering the urban area of Wuhan, China. The images were acquired using the Gaofen-1 and ZY-3 satellite sensors and processed through image fusion and resampling. The dataset contains a total of 4940 RGB images, each with a spatial size of 256×256 pixels and a spatial resolution of approximately 2 m. Pixel-level annotations are provided for six land-cover categories, namely “Bare Soil”, “Building”, “Pavement”, “Road”, “Vegetation”, and “Water”. These categories contain both artificial structures and natural land-cover types, resulting in considerable differences in appearance, texture, shape, and spatial distribution among different classes.

For the experiments in this work, the 4940 images in WHDLD were randomly divided into training, validation, and test sets according to a ratio of 8:1:1. Specifically, 3952 images were used for training, 494 images for validation, and the remaining 494 images for testing. Because the split is performed randomly at the image level rather than according to geographically disjoint regions, strict geographic independence among the training, validation, and test subsets cannot be guaranteed. Compared with the COCO2017-based ball-sport dataset and the UAV-based UDD6 dataset, WHDLD presents substantially different imaging viewpoints, spatial resolutions, object distributions, and scene structures. In particular, urban land-cover categories such as roads, pavements, buildings, and bare soil exhibit relatively similar spectral and visual characteristics, while vegetation and water regions show considerable variations in shape and spatial extent. Therefore, WHDLD provides an additional evaluation of the proposed model in terms of land-cover discrimination, multi-scale feature representation, boundary preservation, and cross-scene applicability under substantially different imaging conditions.

### 3.2. Experimental Settings

The proposed MAF-Net was implemented using PyTorch 2.4.1. For the training and evaluation of MAF-Net, the input images from the COCO2017-derived ball-sports dataset and UDD6 were resized to 640×640 while preserving their original aspect ratios using a letterbox operation, whereas the WHDLD images were processed at their native resolution of 256×256. Bilinear interpolation was adopted for image resizing, whereas nearest-neighbor interpolation was used for the corresponding segmentation masks. The input images were normalized using the ImageNet mean [0.485,0.456,0.406] and standard deviation [0.229,0.224,0.225].

During the training of MAF-Net, simple data augmentation strategies were employed, including random horizontal flipping with a probability of 0.5 and random brightness adjustment with a probability of 0.3. The brightness factor was randomly sampled within the range of [0.85,1.15].

The Adam optimizer was used to optimize the proposed model, with an initial learning rate of $1\times 10^{-3}$. The learning rate was progressively decayed using a polynomial learning-rate schedule with a power of 0.9. MAF-Net was trained for 50 epochs with a batch size of 2. Gradient clipping with a maximum norm of 1.0 was applied to improve training stability. To evaluate the robustness of the results, five independent training runs were conducted for all compared methods, including MAF-Net and all baseline models, using random seeds 42, 2023, 2024, 2025, and 3407. The same training, validation, and test splits were used for every method and every run, while model-specific architectures and training configurations followed their official implementations or the settings recommended in the corresponding publications. For each run, the checkpoint achieving the highest mIoU on the validation set was selected for evaluation on the test set. The quantitative results are reported as the mean ± standard deviation over the five independent runs.

For YOLO11m-seg, the original network architecture was kept unchanged and the model was retrained on each experimental dataset. Semantic annotations were converted into YOLO-compatible polygon labels when necessary. During evaluation, the predicted instance masks were merged into pixel-wise semantic maps and evaluated using the same semantic segmentation metrics as the other methods.

### 3.3. Quantitative and Qualitative Evaluation

To comprehensively evaluate segmentation accuracy and structural preservation across heterogeneous scene types, quantitative and qualitative comparisons are conducted on the COCO2017-derived ball-sports dataset, UDD6, and WHDLD. The proposed method is compared with representative CNN-based, Transformer-based, and lightweight semantic segmentation models, including UPerNet, DeepLabV3, Mask2Former, YOLO11m-seg, TinyViM-S, and PMSFF. All competing methods were reproduced using their official GitHub implementations when available, or otherwise following the hyperparameters and training settings reported in the original papers. The same training, validation, and test splits were adopted for all methods, and inference speed was measured on the same NVIDIA GeForce RTX 4060 GPU with an input resolution of 640×640. Overall accuracy (OA), mean Intersection over Union (mIoU), per-class accuracy, mean Boundary IoU (mBIoU), and small-object class IoU (SC-IoU) are adopted as the main quantitative evaluation metrics. In particular, mBIoU measures the overlap between the predicted and ground-truth boundary regions to evaluate boundary localization and structural preservation. For small-object evaluation, we use a class-based proxy denoted as small-object class IoU (SC-IoU), defined as:

(15) $$\mathrm{SC}\text{-}{\mathrm{IoU}}_{c}=\frac{TP_{c}}{TP_{c}+FP_{c}+FN_{c}},$$

where *c* denotes the selected representative small-object class. Specifically, *c* is the Ball class for the COCO2017-derived ball-sports dataset and the Vehicle class for UDD6. SC-IoU is computed at the pixel level for the selected semantic class and does not apply an explicit instance-size or pixel-area threshold. Therefore, it should be interpreted as a class-based proxy for representative small-object categories rather than a size-stratified small-object metric. For WHDLD, no separate SC-IoU is reported because the object-scale distributions among its semantic categories are relatively similar.

#### 3.3.1. Results on the COCO2017-Derived Ball-Sports Dataset

The quantitative results on the COCO-derived dataset are reported in Table 1. Overall, the proposed method achieves the best performance in terms of both OA and mIoU, while also maintaining competitive or superior performance across all semantic categories. In particular, the relatively high per-class accuracies for Athlete, Equipment, and Ball further indicate competitive performance across different foreground categories.

**Figure 3:**
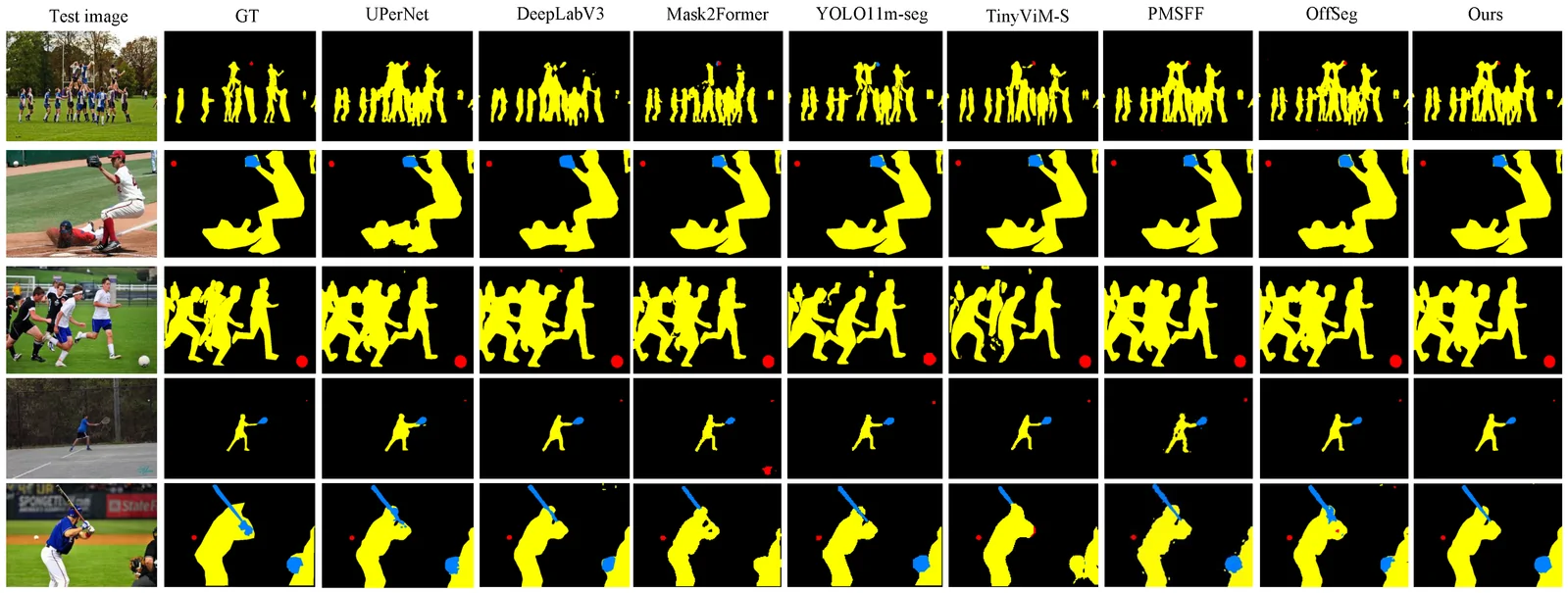
Qualitative comparison of different semantic segmentation methods on the COCO-derived dataset. From left to right: test image, ground truth, UPerNet, DeepLabV3, Mask2Former, YOLO11m-seg, TinyViM-S, PMSFF, OffSeg, and Ours. Yellow, blue, red, and black represent athletes, sports equipment, balls, and background, respectively.

The qualitative results in Figure 3 provide a more intuitive comparison of the segmentation quality of different methods. The three examples cover challenging ball-sport scenes containing crowded athletes, small ball targets, sports equipment, body overlap, and complex object boundaries. These characteristics make accurate segmentation difficult, especially when foreground objects are spatially close to each other.

In the first example, multiple athletes are densely distributed and partially occluded. UPerNet and DeepLabV3 can identify the main human regions, but some local structures are merged or incompletely recovered. Mask2Former and YOLO11m-seg provide better foreground localization, although irregular boundaries and local omissions are still visible. TinyViM-S preserves most of the athlete regions but introduces several local shape deviations. In contrast, the proposed method generates more complete human silhouettes and maintains better separation between adjacent athletes, producing a result that is visually closer to the ground truth.

The second example provides a qualitative illustration of the model’s behavior on relatively small foreground regions. In addition to the athlete regions, the scene contains a very small ball and sports equipment occupying only a limited number of pixels. Some comparison methods either weaken these small regions or produce inaccurate contours around the foreground objects. The proposed method preserves the ball and equipment more clearly while maintaining the overall integrity of the athlete regions. This qualitative observation suggests that useful local spatial information is retained during feature extraction and reconstruction.

In the third example, several athletes appear close to each other together with a small ball target. The comparison methods generally recover the dominant human regions, but differences become apparent around overlapping body parts and object boundaries. Overall, the shown examples suggest that the proposed method produces spatially coherent segmentation results across scenes containing objects with different scales and structures.

Overall, the visual comparisons show that the proposed method is not only effective in recognizing large foreground regions but also provides better structural preservation for small objects, slender sports equipment, and occluded athletes. The predicted masks exhibit fewer fragmented regions and more consistent object contours.

#### 3.3.2. Results on the UDD6 Dataset

The quantitative comparison on the UDD6 dataset is presented in Table 2. The proposed method achieves the best overall OA and mIoU among the compared models and shows consistently strong performance across most semantic categories. These results show that the performance gains observed on the ball-sports dataset are largely maintained in aerial urban scenes with markedly different object distributions, viewpoints, and spatial structures. This provides further evidence that the proposed morphology-aware feature modeling strategy is not limited to a specific application scenario and exhibits good cross-scene applicability.

**Figure 4:**
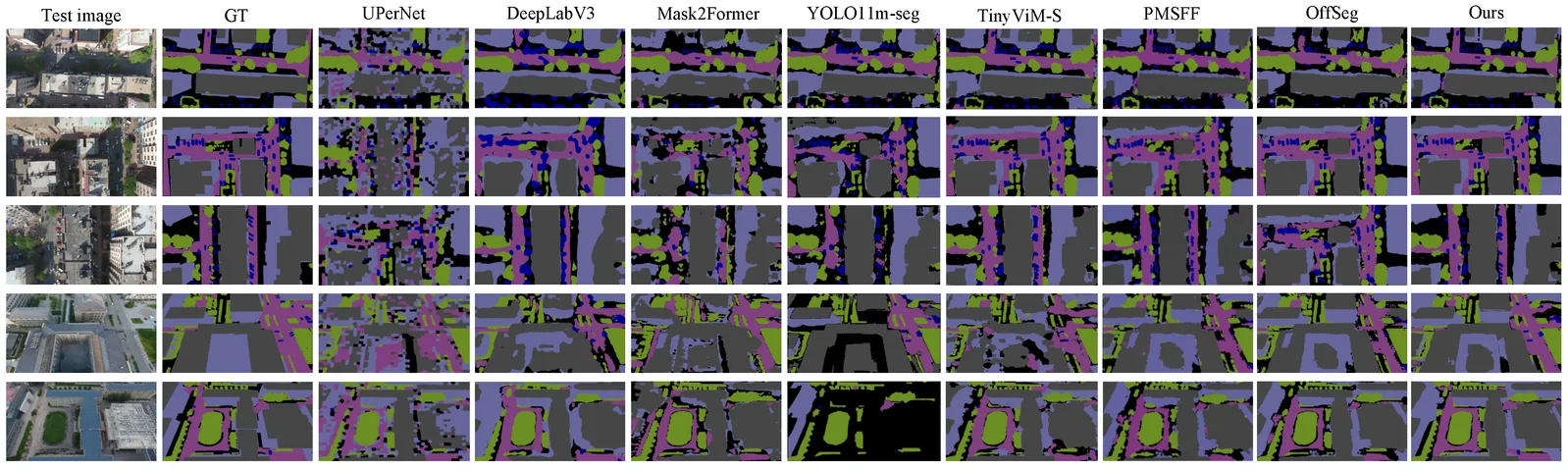
Qualitative comparison of different semantic segmentation methods on the UDD6 dataset. From left to right: test image, ground truth, UPerNet, DeepLabV3, Mask2Former, YOLO11m-seg, TinyViM-S, PMSFF, OffSeg, and Ours. Black, grayish purple, purple, olive green, dark blue, and dark gray represent Other, Facade, Road, Vegetation, Vehicle, and Roof, respectively.

As shown in Figure 4, the UDD6 dataset presents a different type of segmentation challenge from COCO2017. The aerial images contain buildings, roads, vegetation, vehicles, and other urban structures with large variations in scale and shape. Moreover, many semantic regions are adjacent to each other and form complex spatial layouts, making both regional consistency and boundary localization important.

In the first example, the urban scene contains a relatively complex arrangement of buildings, vegetation, and surrounding regions. UPerNet produces noticeable fragmentation and scattered predictions in several areas, indicating difficulty in maintaining spatial consistency. DeepLabV3 improves the overall region continuity but still generates relatively coarse transitions between neighboring classes. Mask2Former, YOLO11m-seg, and TinyViM-S provide more stable predictions, but local category confusion remains around complex boundaries. The proposed method generates more compact semantic regions and preserves the overall spatial layout more consistently with the ground truth.

The second example contains several elongated and narrow structures distributed between larger semantic regions. This scene is particularly challenging because small boundary errors can significantly alter the shape of these structures. Some comparison methods show discontinuities, local misclassification, or excessive merging of adjacent regions. In the shown example, the proposed method produces fewer visible discontinuities in several narrow regions while maintaining relatively clear separation between neighboring semantic categories.

A similar tendency can be observed in the third example, where multiple elongated regions and irregular object boundaries appear simultaneously. UPerNet shows relatively strong fragmentation, whereas the other comparison methods recover the main structures but still exhibit local boundary distortion or category confusion. The segmentation produced by the proposed model is more spatially coherent and follows the ground-truth structure more closely, especially along long boundaries and narrow regions.

These visual results suggest that the proposed method is able to maintain both local boundary details and global regional consistency. More importantly, the improvements are observed not only in ball-sport images but also in aerial urban scenes with substantially different visual characteristics. This demonstrates that the proposed feature modeling strategy has good adaptability to objects with diverse scales, shapes, and spatial arrangements.

#### 3.3.3. Results on the WHDLD Dataset

As shown in Table 3, the proposed MAF-Net achieves the best performance in terms of OA and mIoU on the WHDLD dataset, reaching 84.24±0.57% and 63.94±0.26%, respectively. Compared with the second-best OffSeg, MAF-Net improves OA and mIoU by 0.39 and 0.78 percentage points, respectively. In terms of boundary segmentation, MAF-Net achieves an mBIoU of 50.06±0.45%, which is comparable to the best result of 50.13±0.74% obtained by OffSeg. These results demonstrate that MAF-Net maintains competitive segmentation accuracy and boundary preservation performance on remote-sensing scenes.

**Figure 5:**
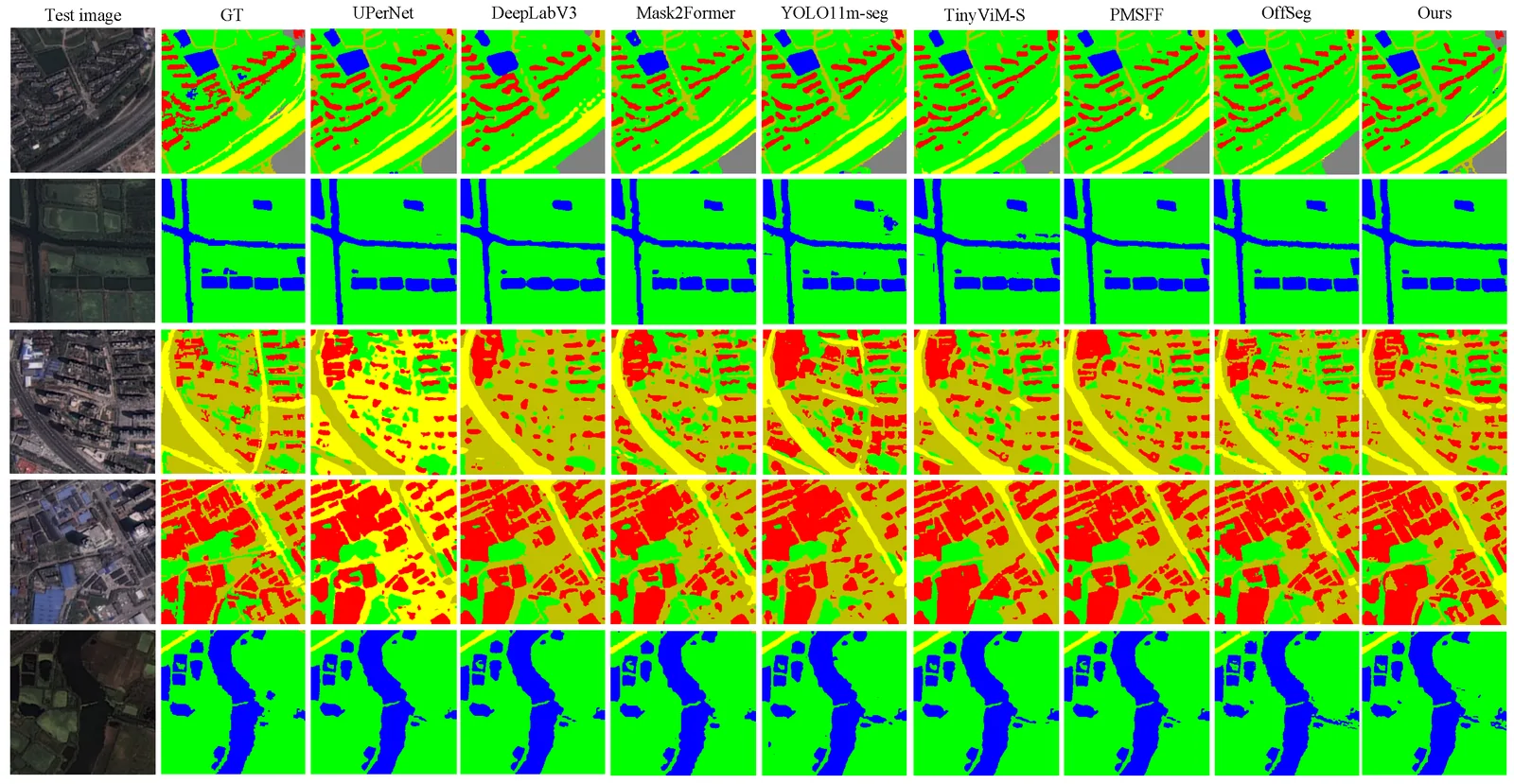
Qualitative comparison of different semantic segmentation methods on the WHDLD dataset. From left to right: test image, ground truth, UPerNet, DeepLabV3, Mask2Former, YOLO11m-seg, TinyViM-S, PMSFF, OffSeg, and Ours. Gray, red, olive, yellow, green, and blue represent Bare Soil, Building, Pavement, Road, Vegetation, and Water, respectively.

The qualitative comparisons in Figure 5 further show that MAF-Net preserves relatively clear land-cover boundaries and local structural details in complex remote-sensing scenes. Despite the visual similarity between some neighboring land-cover categories, the proposed method produces spatially coherent segmentation results with relatively well-preserved region structures. These qualitative observations, together with the quantitative results, further support the effectiveness and cross-scene applicability of MAF-Net for semantic segmentation.

## 4. Discussion

### 4.1. Inference Efficiency and Model Complexity

In addition to segmentation accuracy, the computational efficiency and model complexity of different methods are also evaluated. The reported GFLOPs are calculated for a single forward pass with an input resolution of 640×640. Only the operations within the network forward process are included, while image preprocessing and post-processing operations, such as resizing the prediction to the original image resolution, Softmax, argmax, and color mapping, are excluded.

As shown in Table 4, MAF-Net achieves the highest inference speed of 60.36 FPS and the smallest parameter size of 4.65 M among all compared methods. Its computational complexity is 29.45 GFLOPs, ranking second lowest among the evaluated models. This indicates that MAF-Net can maintain a low computational cost while providing high practical inference efficiency. Combined with its competitive segmentation accuracy, these results demonstrate a good balance among accuracy, model size, computational complexity, and inference speed.

### 4.2. Ablation Study

To evaluate the contribution of each component, we conducted ablation studies on the COCO-derived, UDD6, and WHDLD datasets using a fixed random seed of 42.

#### 4.2.1. Ablation of Network Components

To evaluate the contribution of each component, we conducted ablation studies on the COCO-derived, UDD6, and WHDLD datasets. The results are shown in Table 5. We evaluated MAKD, the kernel design, the fusion strategy, and TFFRM.

The complete MAF-Net achieves the best performance on all three datasets. When adaptive gating is replaced by average fusion, the mIoU decreases from 79.23% to 75.02% on the COCO-derived dataset, from 73.18% to 68.78% on UDD6, and from 64.13% to 63.27% on WHDLD. The decreases are 4.21, 4.40, and 0.86 percentage points, respectively. This shows that adaptive gating can better combine features from different branches.

We also replaced the heterogeneous kernel configuration (7×7, 3×3, 1×7, and 7×1) with four 3×3 kernels. The mIoU decreases by 2.53, 1.34, and 1.33 percentage points on the COCO-derived, UDD6, and WHDLD datasets, respectively. This result shows that kernels with different sizes and directions provide useful complementary features.

Removing MAKD decreases the mIoU by 4.97, 3.06, and 2.48 percentage points on the three datasets, respectively. Removing TFFRM decreases the mIoU by 3.78, 3.15, and 1.07 percentage points, respectively. These results show that both MAKD and TFFRM contribute to the final performance. Overall, the ablation results verify the effectiveness of the proposed components.

**Figure 6:**
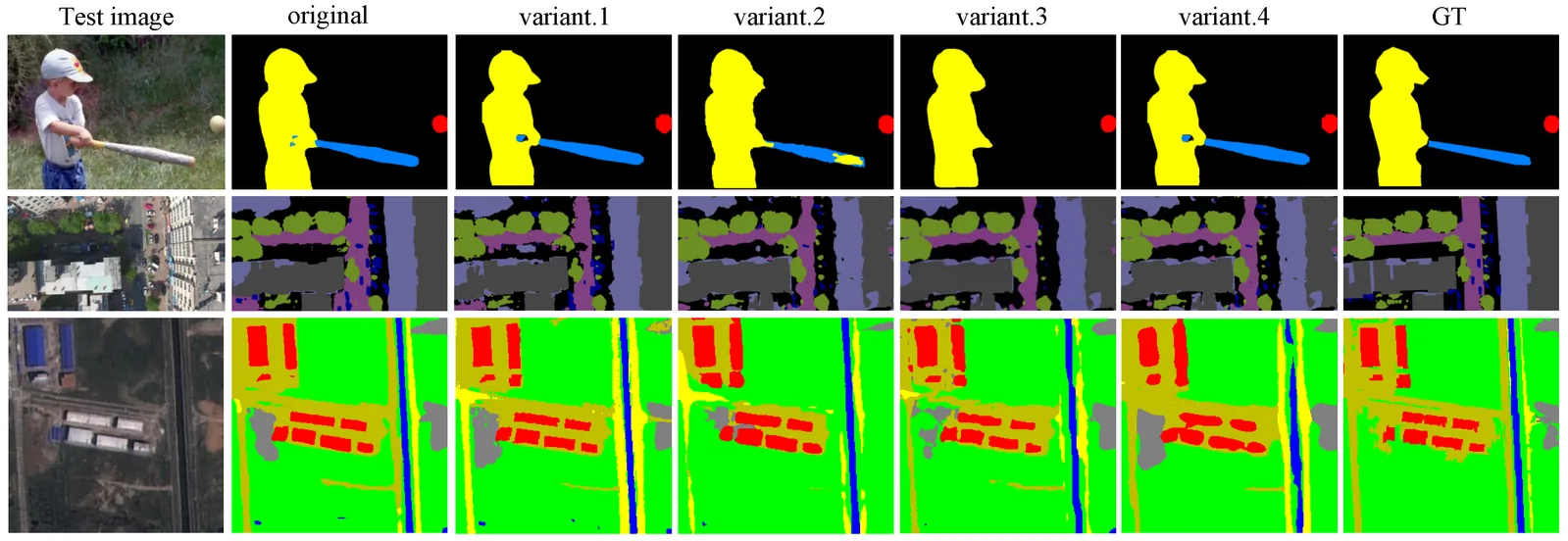
Visualization of different variants of ablation experiments.

In addition to the quantitative analysis, Figure 6 provides qualitative comparisons of the complete model and different ablation variants. The ablated variants exhibit more local misclassification, structural discontinuities, or boundary degradation in challenging regions, whereas the complete MAF-Net produces more coherent segmentation results that are closer to the ground truth. These visual comparisons further support the effectiveness of the proposed components.

#### 4.2.2. Sensitivity Analysis of Batch Size

**Figure 7:**
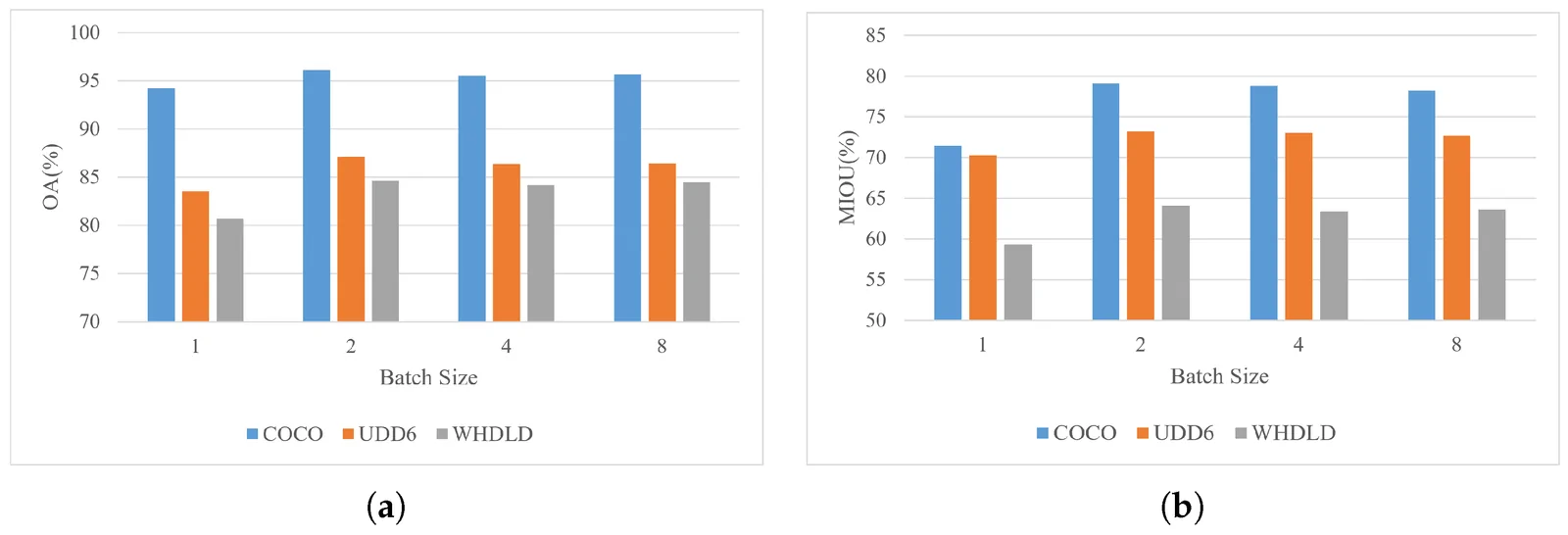
Sensitivity analysis of batch size on the COCO-derived, UDD6, and WHDLD datasets. (**a**) OA under different batch sizes; (**b**) mIoU under different batch sizes.

To further investigate the influence of batch size on training stability and segmentation performance, we conducted a sensitivity analysis using batch sizes of 1, 2, 4, and 8 while keeping the other training settings unchanged. As shown in Figure 7, a batch size of 2 achieves the best overall performance on the COCO-derived, UDD6, and WHDLD datasets in terms of both OA and mIoU. Increasing the batch size to 4 or 8 results in only small performance variations, whereas a batch size of 1 leads to a more noticeable degradation. These results indicate that the model is relatively stable across different batch sizes and support the choice of a batch size of 2 in our experiments.

#### 4.2.3. Effect of Native Input Resolution on WHDLD

Since the original WHDLD images have a resolution of 256×256, we further evaluate MAF-Net using the native image resolution to investigate the trade-off between segmentation accuracy and computational efficiency. For this experiment, the model was retrained and evaluated at 256×256 using the same training settings as the 640×640 configuration, with only the input resolution changed. The results are summarized in Table 6.

As shown in Table 6, reducing the input resolution from 640×640 to the native 256×256 resolution results in only a slight performance decrease, with OA, mIoU, and mBIoU dropping by 0.36, 0.58, and 0.48 percentage points, respectively. In contrast, the computational cost is substantially reduced from 29.45 to 14.91 GFLOPs, while the inference speed increases from 60.36 to 180.54 FPS, nearly three times faster. These results indicate that using the native resolution provides a more favorable accuracy–efficiency trade-off on WHDLD, especially for resource-constrained deployment.

### 4.3. Limitations and Failure Case Analysis

**Figure 8:**
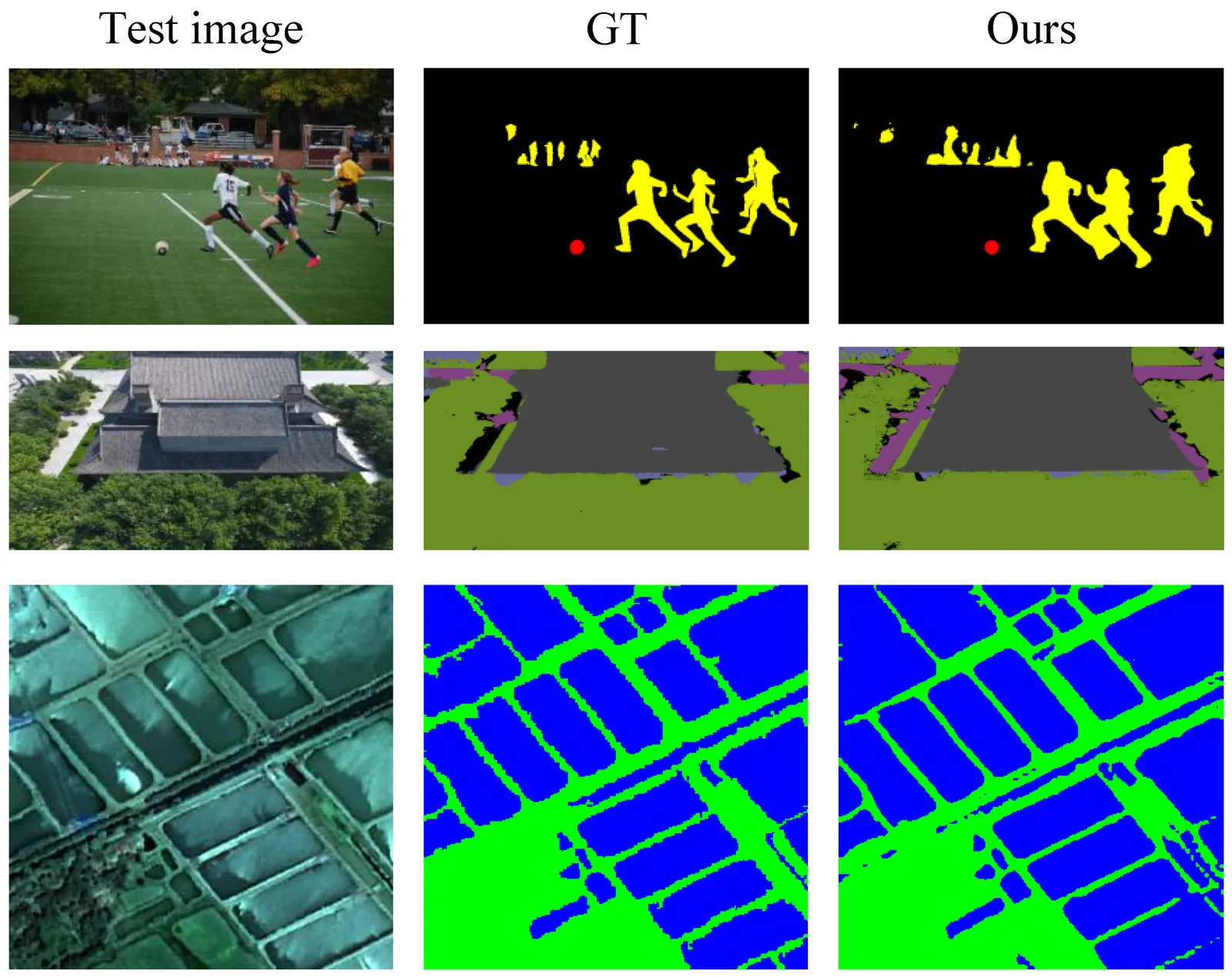
Representative failure cases of MAF-Net on challenging samples. From **left** to **right**: test image, ground truth, and prediction of MAF-Net.

Although MAF-Net achieves competitive segmentation performance across different scenes, some challenging cases remain. Figure 8 presents several representative failure examples from the three datasets.

As shown in Figure 8, the first example contains small foreground targets and partially occluded objects. Although the main object regions are correctly identified, some small or heavily occluded regions are still missed or incompletely segmented. This indicates that extremely small targets remain challenging when their visual information is limited or disturbed by nearby objects.

In the aerial and remote-sensing examples, local category confusion can still occur between adjacent regions with similar appearance. In addition, thin structures and complex boundaries are occasionally over-smoothed or locally misclassified, leading to slight deviations from the ground truth. These cases suggest that, although MAKD and TFFRM improve multi-scale representation and structural refinement, the model still has limitations in preserving very fine structures and distinguishing visually similar neighboring categories.

Future work will therefore focus on improving fine-grained feature representation for extremely small and occluded objects, introducing stronger boundary-aware constraints, and enhancing inter-class discrimination in visually ambiguous regions while maintaining the lightweight design of MAF-Net.

Another limitation concerns the random split of the WHDLD dataset. Since the dataset is randomly divided at the image level, similar samples may appear in the training, validation, and test subsets. This may result in slightly favorable performance estimates on WHDLD. In future work, more rigorous data partitioning strategies will be considered to further evaluate the robustness of the proposed method.

## 5. Conclusions

To address morphology heterogeneity caused by substantial variations in object scale, geometric structure, and boundary characteristics in semantic segmentation, this paper proposes a lightweight morphology-adaptive segmentation network, termed MAF-Net. The model adopts MobileNetV3-Large as the backbone and integrates multi-scale features through a top-down encoding structure. A Morphology-Adaptive Kernel Decoder is introduced to combine convolutional branches with different receptive-field scales and directional sensitivities through spatially adaptive gating, while a Triple Feature Fusion Refinement Module integrates decoded semantic features, shallow spatial details, and implicitly learned boundary-aware responses for structural refinement.

Experiments on three datasets with substantially different scene characteristics (the COCO-derived, UDD6, and WHDLD datasets) show that MAF-Net achieves consistently competitive segmentation performance across sports, aerial urban, and remote-sensing scenes. Meanwhile, the model requires only 4.65 M parameters and achieves an inference speed of 60.36 FPS under the reported experimental setting. The results indicate that the proposed morphology-aware design is not restricted to a specific scene type and provides a favorable balance between segmentation accuracy and computational efficiency across heterogeneous segmentation scenarios. The ablation results further demonstrate the positive contributions of the morphology-adaptive decoder, adaptive gating mechanism, heterogeneous kernel configuration, and refinement module.

Future work can be further explored in several directions. First, the perception capability for small targets and fine-grained structures can be further improved by introducing multi-scale supervision, boundary constraints, or more refined feature enhancement strategies, thereby enhancing the recognition of small-scale objects and targets with complex geometric structures. In addition, lightweight Transformer architectures or state space models can be investigated to strengthen the modeling of long-range spatial dependencies and global contextual information while controlling computational overhead, further improving semantic segmentation performance in complex scenes.

## Figures and Tables

**Table 1:** Quantitative comparison of different methods on the COCO2017-derived ball-sports dataset. Results are reported as mean ± standard deviation over five runs. (The optimal results are highlighted in bold).

Methods	OA (%)	mIoU (%)	mBIoU (%)	SC-IoU (%)
UPerNet [15]	90.75±1.13	63.25±0.93	53.80±1.14	39.71±0.69
DeepLabV3 [13]	92.09±1.54	66.96±0.89	54.91±1.55	43.74±0.64
Mask2Former [19]	92.08±0.98	65.50±1.07	57.96±1.11	48.85±1.10
YOLO11m-seg [35]	93.42±1.24	73.80±1.00	61.02±0.79	51.02±1.03
TinyViM-S [36]	95.24±0.48	74.12±0.38	61.38±0.42	52.25±0.38
PMSFF [43]	95.04±0.51	77.50±0.32	63.91±0.72	53.99±0.40
OffSeg [44]	95.30±0.37	75.76±0.40	63.19±0.44	52.91±0.75
Ours	96.06±0.26	79.80±0.42	66.33±0.34	55.21±0.64

**Table 2:** Quantitative comparison of different methods on the UDD6 dataset. Results are reported as mean ± standard deviation over five runs. (The optimal results are highlighted in bold).

Methods	OA (%)	mIoU (%)	mBIoU (%)	SC-IoU (%)
UPerNet [15]	66.60±0.90	44.49±0.96	41.47±0.41	44.42±0.63
DeepLabV3 [13]	78.05±0.85	58.87±1.03	48.17±0.62	49.06±0.48
Mask2Former [19]	74.66±0.80	60.98±0.72	48.01±0.55	49.89±0.67
YOLO11m-seg [35]	83.09±0.43	64.82±0.91	50.03±0.47	50.75±0.71
TinyViM-S [36]	84.97±0.52	69.65±0.76	51.80±1.04	52.55±0.41
PMSFF [43]	87.16±0.69	71.19±0.58	53.89±0.88	54.63±0.77
OffSeg [44]	82.06±0.75	66.72±1.56	50.95±0.43	51.56±1.10
Ours	86.79±0.55	72.48±0.60	55.98±0.40	56.95±0.57

**Table 3:** Quantitative comparison of different methods on the WHDLD dataset. Results are reported as mean ± standard deviation over five runs. (The optimal results are highlighted in bold).

Methods	OA (%)	mIoU (%)	mBIoU (%)
UPerNet [15]	79.86±0.59	55.90±1.17	45.53±0.76
DeepLabV3 [13]	81.17±0.67	60.20±0.43	48.38±0.69
Mask2Former [19]	83.30±0.58	61.51±0.52	48.39±0.62
YOLO11m-seg [35]	80.83±0.62	60.87±0.51	49.12±0.56
TinyViM-S [36]	81.60±0.72	59.49±0.83	47.48±0.79
PMSFF [43]	82.08±0.49	61.64±0.50	49.12±0.27
OffSeg [44]	83.85±0.50	63.16±0.43	50.13±0.74
Ours	84.24±0.57	63.94±0.26	50.06±0.45

**Table 4:** Comparison of inference efficiency and model complexity. (The optimal results are highlighted in bold).

Methods	Input Size	FPS (img/s)	Param (M)	GFLOPs
UPerNet [15]	640×640	22.93	8.69	52.02
DeepLabV3 [13]	640×640	27.32	42.32	270.83
Mask2Former [19]	640×640	24.58	36.24	93.27
YOLO11m-seg [35]	640×640	44.79	20.12	108.71
TinyViM-S [36]	640×640	51.22	5.68	15.38
PMSFF [43]	640×640	38.54	5.46	33.64
OffSeg [44]	640×640	54.54	6.16	44.37
Ours	640×640	60.36	**4.65**	29.45

**Table 5:** Results of the ablation study on the COCO-derived, UDD6, and WHDLD datasets. (✓ indicates the component is included, × indicates it is excluded.)

MAKD	Kernel Design	Fusion Strategy	TFFRM	COCO-Derived		UDD6		WHDLD	
				OA	mIoU	OA	mIoU	OA	mIoU
✓	7×7,3×3,1×7,7×1	Adaptive Gate	✓	96.11	79.23	87.08	73.18	84.64	64.13
✓	7×7,3×3,1×7,7×1	Average Fusion	✓	93.77	75.02	82.37	68.78	83.74	63.27
✓	3×3,3×3,3×3,3×3	Adaptive Gate	✓	95.26	76.70	85.61	71.84	83.44	62.80
×	None	None	✓	93.79	74.26	84.19	70.12	82.32	61.65
✓	7×7,3×3,1×7,7×1	Adaptive Gate	×	94.44	75.45	84.43	70.03	83.62	63.06

**Table 6:** Effect of input resolution on MAF-Net for the WHDLD dataset.

Input Size	OA (%)	mIoU (%)	mBIoU (%)	FPS	Param (M)	GFLOPs
640×640	84.64	64.13	50.60	60.36	4.65	29.45
256×256	84.28	63.55	50.12	180.54	4.65	14.91

## Data Availability

The data presented in this study are available upon request from the corresponding author.

## Abbreviations

The following abbreviations are used in this manuscript:

MAF-Net	Morphology-Adaptive Feature Fusion Network
MAKD	Morphology-Adaptive Kernel Decoder
TFFRM	Triple Feature Fusion Refinement Module
OA	Overall Accuracy
mIoU	mean Intersection over Union
CRF(s)	Conditional Random Field(s)
ASPP	Atrous Spatial Pyramid Pooling
UAV	Unmanned Aerial Vehicle
UDD	Urban Drone Dataset
WHDLD	Wuhan Dense Labeling Dataset
DWConv	Depthwise Convolution
PWConv	Pointwise Convolution
CNN	Convolutional Neural Network
FCN	Fully Convolutional Network
COCO2017	Common Objects in Context (2017)
GFLOPs	Giga Floating-point Operations
FPS	Frames Per Second
